# Supplementary figures and images for: Transcriptome analysis reveals the mechanism of improving erect-plant-type peanut yield by single-seeding precision sowing
Source: PeerJ. 2021 Feb 9;9:e10616. doi: 10.7717/peerj.10616 (PMC7879956; doi:10.7717/peerj.10616)

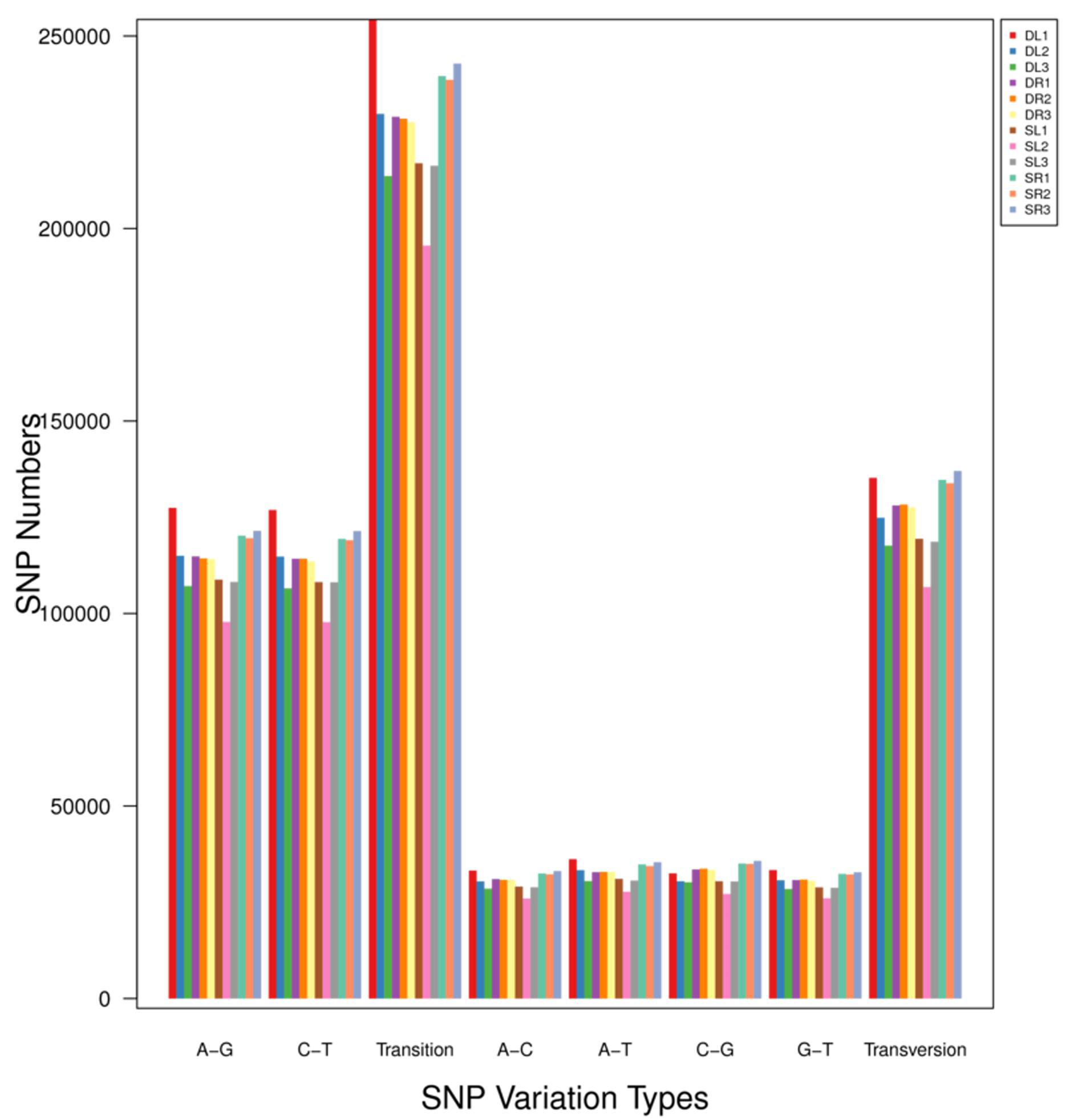

Supplement: Supplemental Information 1 — A–G: The amount of A–G variant type; C–T: The amount of C–T variant type; Transition: The amount of A–G and C–T variant type; A–C: The amount of A–C variant type; A–T: The amount of A–T variant type; C–G: The amount of C–G variant type; G–T: The amount of G–T variant type; Transversion: The amount of A–C, A–T, C–G and G–T variant type. [file peerj-09-10616-s001.png]

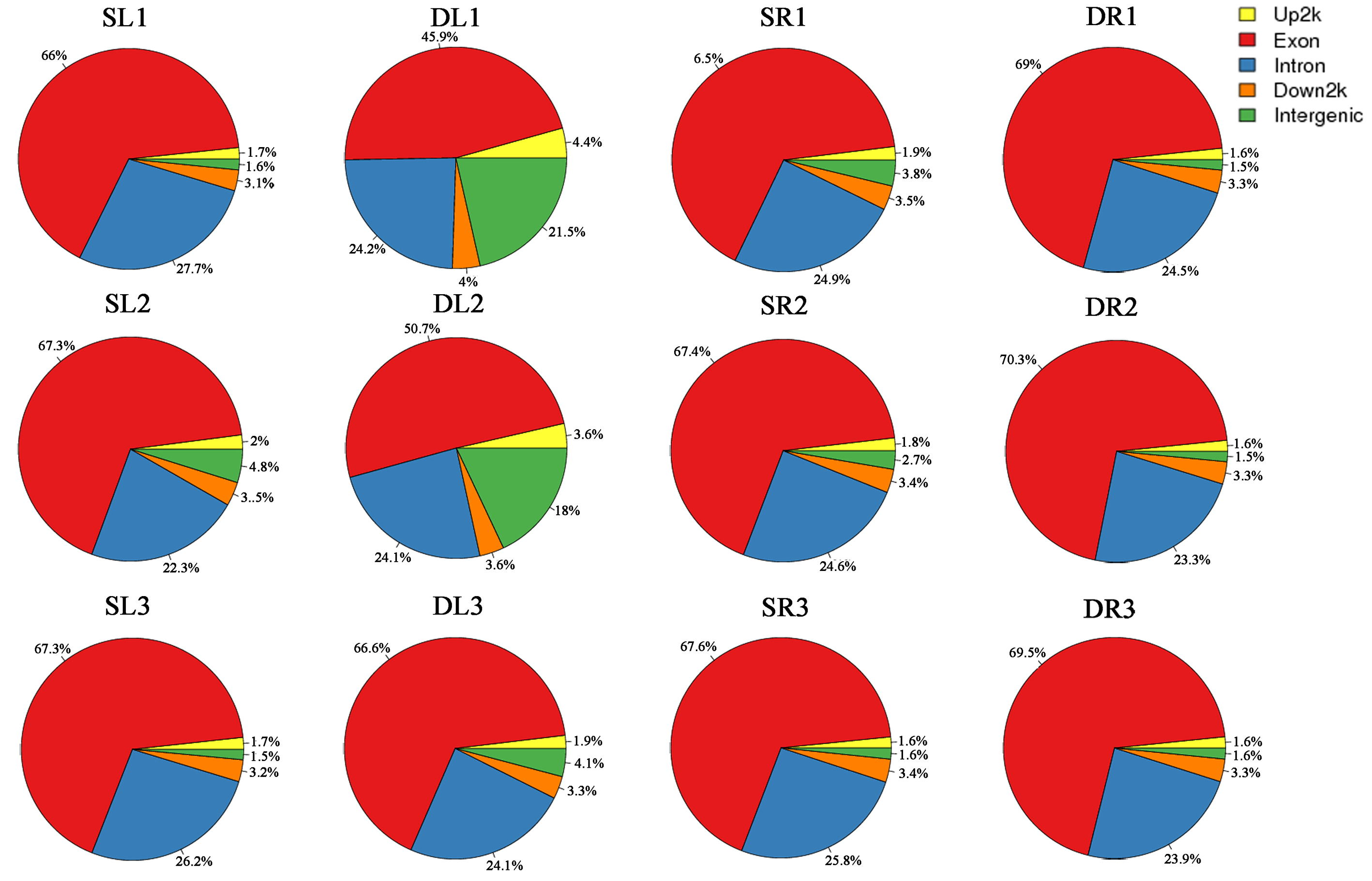

Supplement: Supplemental Information 2 — Up2k means upstream 2,000 bp area of a gene. Down2k means downstream 2,000 bp area of a gene. [file peerj-09-10616-s002.png]

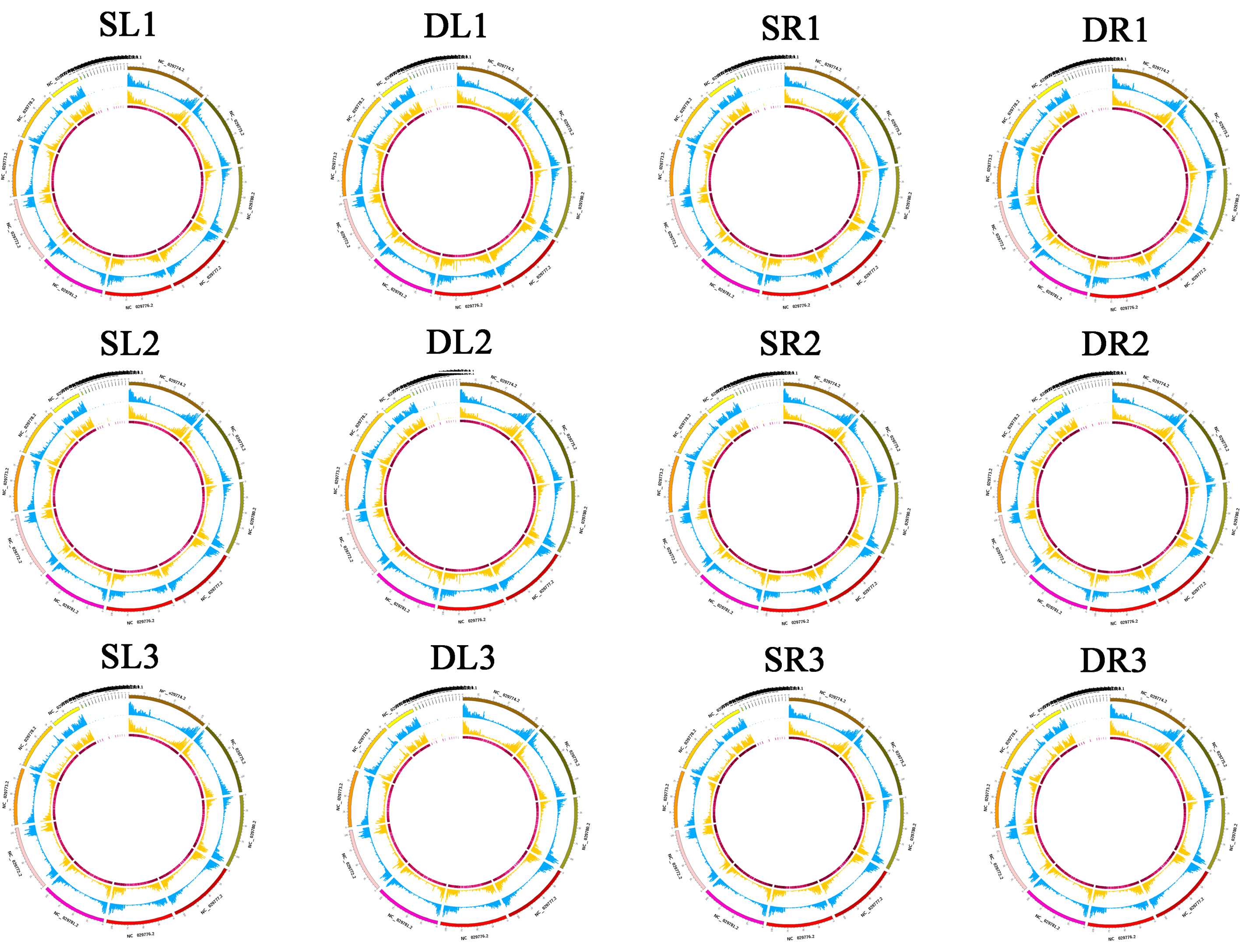

Supplement: Supplemental Information 3 [file peerj-09-10616-s003.png]

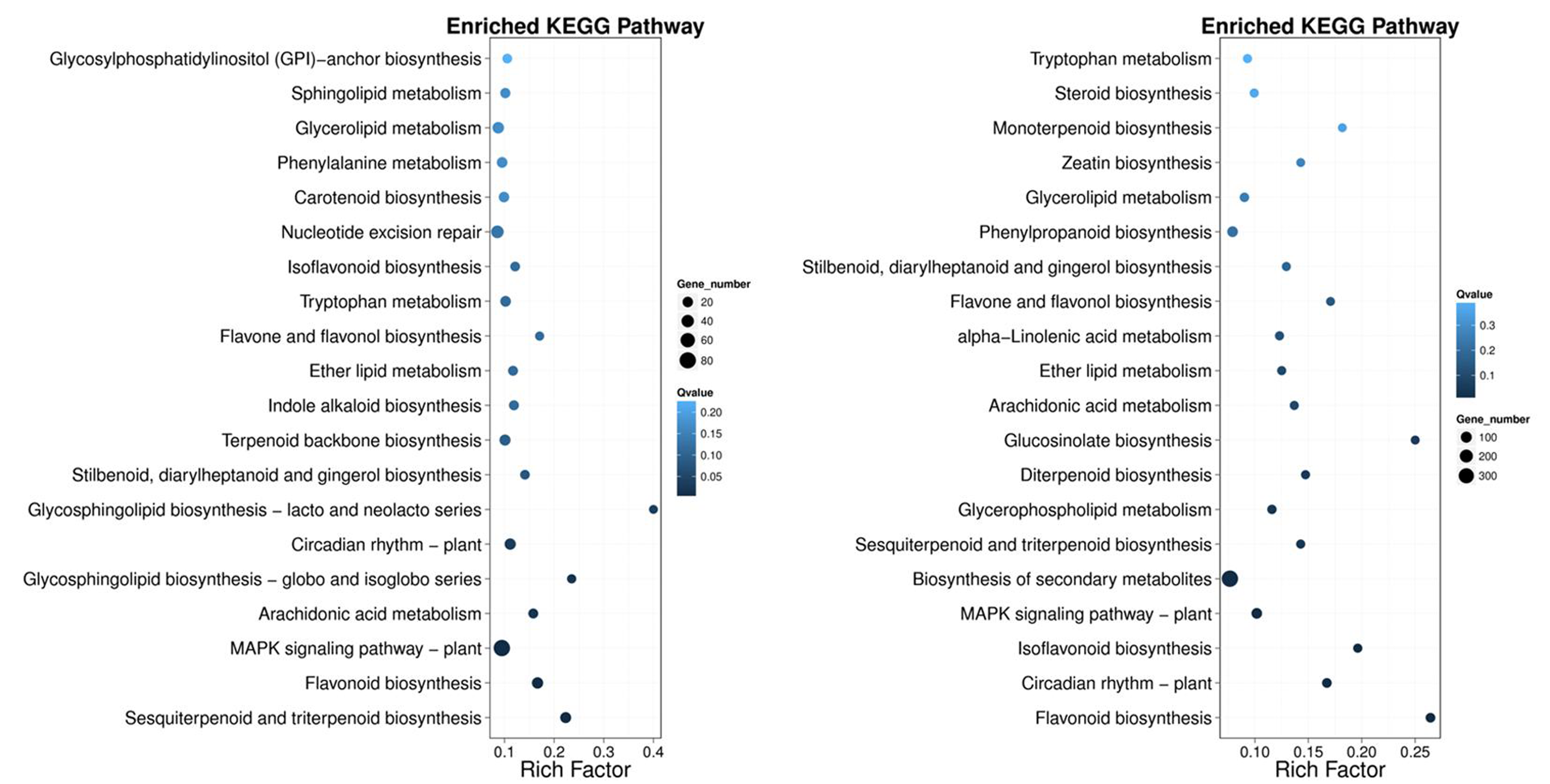

Supplement: Supplemental Information 4 — X axis represents enrichment factor. Y axis represents pathway name. The color indicates the q-value (high: white, low: blue), the lower q-value indicates the more significant enrichment. Point size indicates DEG number (The bigger dots refer to larger amount). Rich Factor refers to the value of enrichment factor, which is the quotient of foreground value (the number of DEGs) and background value (total Gene amount). [file peerj-09-10616-s004.png]
