# Supplementary material for: Transcriptome analysis reveals the mechanism of improving erect-plant-type peanut yield by single-seeding precision sowing
Source: PeerJ. 2021 Feb 9;9:e10616. doi: 10.7717/peerj.10616 (PMC7879956; doi:10.7717/peerj.10616)
Supplement: Supplemental Information 5 — The data presented are the mean values ± SD of three individual experiments. [file peerj-09-10616-s005.docx]

Table S1 Variety of the activities of SOD, POD, CAT, and TTC in SS and DS sowing treatments, respectively. The data presented are the mean values ± SD of three individual experiments.

| Treatment | SOD /(U·g^-1^FW) | POD /(△470·g^-1^) | CAT /(mg·g^-1^·min^-1^) | TTC (μg·g^-1^·FW·h^-1^) |
| --- | --- | --- | --- | --- |
| SS | 168.32a | 132.47a | 32.56a | 56.94a |
| DS | 135.45b | 112.87b | 25.43b | 41.38b |
